# Salvage Strategies for Local Recurrences of Squamous Cell Carcinoma of the Nasal Vestibule: A Single-Center Experience of 22 Years

**DOI:** 10.3390/jcm13020541

**Published:** 2024-01-18

**Authors:** Corrado Rubino, Emilio Trignano, Francesco Bussu, Pietro Luciano Serra, Manuela Rodio, Matilde Tettamanzi, Silvia Rampazzo

**Affiliations:** 1 Plastic Surgery Unit, University Hospital Trust of Sassari, Viale S. Pietro 43, 07100 Sassari, Italy; corubino@uniss.it (C.R.); etrignano@uniss.it (E.T.); m.rodio@studenti.uniss.it (M.R.); m.tettamanzi@studenti.uniss.it (M.T.); 2 Department of Medicine, Surgery and Pharmacy, University of Sassari, Viale S. Pietro 43, 07100 Sassari, Italy; fbussu@uniss.it; 3 Otolaryngology Division, University Hospital Trust of Sassari, Viale S. Pietro 43, 07100 Sassari, Italy; 4 Plastic, Reconstructive and Aesthetic Surgery Training Program, University of Sassari, Viale S. Pietro 43, 07100 Sassari, Italy; p.serra4@studenti.uniss.it

**Keywords:** squamous cell carcinoma, nasal vestibule, recurrence, reconstructive surgery

## Abstract

Squamous cell carcinomas of the nasal vestibule are an extremely rare neoplastic disease. Although brachytherapy is gaining popularity for primary treatment, surgery remains the best option in case of recurrences. The aim of this paper is to outline our treatment experience of local recurrence of SCCNVs over the past 22 years. We retrospectively reviewed the clinical data of the patients who underwent surgical treatment for local recurrence of SCCNV: data regarding age, sex, primary tumor treatment, recurrence location and time of appearance, surgical resection, type of reconstruction, postoperative complication, surgical revision, and re-recurrence rate were analyzed. Twenty patients were included in the study. The median period for recurrence appearance was 17 months, and the prevalent location of recurrence was the nasal alae. Prevalent reconstructive procedures were the nasolabial flap and paramedian forehead flap. No postoperative complications were observed, and one case of re-recurrence was detected at 12-months of follow-up. Based on our experience, salvage surgical procedures for SCCNV recurrences must be individualized and carefully planned, taking into account the peculiar pattern of tumor spread and the presence of scar and heavily radiotherapy damaged tissue from previous treatment; delayed reconstruction should be considered for all the cases with skeletal involvement.

## 1. Introduction

Squamous cell carcinomas originating from the epithelium of the nasal vestibule, referred to as Squamous cell carcinomas of the nasal vestibule (SCCNV), represent and exceedingly rare neoplastic disease. They represent less than 1% of head and neck malignancies [1], but the actual incidence is thought to be higher as they are frequently mistaken as skin primaries. Despite their origin from the epithelium of the nasal vestibule, these carcinomas typically exhibit an early straightforward invasion of the skin. Consequently, if the nasal cavity is not thoroughly evaluated during clinical examination, these tumors are frequently mistaken as skin primaries, requiring multiple surgical excisions to obtain the radical removal of the lesion. Squamous cell carcinomas of the nasal vestibule exhibit distinctive patterns of spread along cartilaginous surfaces based on their original location [2]. In particular, lesions of the interior lining of the ala nasi often spread directly to the skin of the ala passing directly through the fibro-areolar tissue, whereas lesions of the interior lining of the alar cartilages often run superiorly and spread to the skin of the nasal tip passing through the internal nasal valve. Conversely, lesions of the interior lining of the lateral cartilage run along the deep surface of the cartilage until reaching the junction with the nasal and maxillary bones, where they reach the skin passing between cartilage and bone. On the other hand, lesions of the medial wall spread posteriorly along the cartilaginous septum up to the vomer, and/or to the columella and the nasal spine, whereas lesions of the nasal sill spread to the upper lip, nasal spine, and hard palate. Infiltration of cartilaginous portion of the nasal structures is, instead, usually detected in more advanced stages of the disease.

Treatment options for primary squamous cell carcinomas of the nasal vestibule include surgery and radiotherapy, either as standalone modalities or in combination, depending on the stage of the disease [2,3,4,5,6,7,8,9]. Radiotherapy can be either delivered through External Beam (EBRT) or Brachytherapy (BT). Currently there is no conclusive evidence in the literature supporting the superiority of any treatment modality in terms of oncological results for early stage diseases [10,11,12,13,14]. However, a few papers have reported better aesthetic and functional outcomes with BT [12,13,15,16]. For advanced stage tumors, instead, combined treatment in the form of surgery followed by adjuvant radiotherapy is recommended [17,18,19].

Locoregional control after primary treatment is reported to range between 69% and 95% [3,4,5,11], with advanced stages and tumor originating from the septum being associated with a higher incidence of local recurrences [5,20]. Recurrences are documented within 8 to 42 months post-treatment [4,5,9,20]. Due to the rarity of the event, evidence regarding the best treatment option for local recurrences is lacking. Different authors have used RT and surgery as salvage treatment [5,6,20,21], with good locoregional disease control rates. Nevertheless, as worse aesthetic outcomes have been reported with the use of BT in case of recurrences [16], surgery remains the current standard in such cases. The surgical treatment of local recurrences entails unique challenges in terms of surgical demolition and reconstruction due to the complex anatomy of the region, the pattern of recurrence, and the presence of radiation damage or scars derived from the previous treatment of the primary lesion.

The aim of this paper is to report our experience over the past 22 years in the surgical treatment of SCCNV recurrences, describing our surgical approach in relation to the location of such recurrences and provide potential recommendations for the management of these patients.

## 2. Materials and Methods

A retrospective analysis of the patients who underwent surgical treatment for local recurrence of SCCNV in our institution at the University Hospital Trust of Sassari from 2000 to 2022 was performed. Data regarding patients’ sex and age, primary lesion treatment, time for local recurrence appearance, location of the recurrence, preoperative imaging evaluation, type of resection and reconstruction, post-operative complications, and re-recurrence appearance were recorded from the patients’ medical charts. We enrolled all the patients with a minimum follow-up period of 12 months. Clinical evaluation for re-recurrences is routinely performed every 6 months for the first two years, and yearly afterward in our institution. Breathing was evaluated as a patient-reported outcome measure one year after the completion of the reconstruction. 

The data were entered in a Microsoft Excel 2021 sheet. The continuous variables were described as median and range. Discrete variables were expressed as percentages. The main end point analyzed was local recurrence. The Kaplan–Meier method was used to estimate the recurrence-free survival (RFS). Patients were censored at last contact if lost to follow-up or at the date of death due to any cause.

## 3. Results

From 2000 to 2022, 20 patients were surgically treated at our institution for local recurrence of a squamous cell carcinoma of the nasal vestibule. The study group consisted of four females and sixteen males, with a median age of 71 years (range 56–78). Data regarding the local recurrence and the reconstructive surgical procedure are summarized in Table 1. 

The median period for recurrence appearance was 17 months (range 8 to 30 months). The majority of patients (16 out of 20 cases) were treated for a post-surgery recurrence, whereas the remaining part (4 out of 20) were treated for a post-radiotherapy recurrence. It is important to note that the above proportion does not reflect the success of primary treatment, but it does reflect the features of the population included in the study. Incisional biopsy of the suspected lesion was done to histologically confirm the recurrence. Pre-operative radiologic evaluation with CT scan and/or MRI was done in all the patients to better assess soft tissue and/or bone infiltration. Infiltration of the skeletal portion of the nasal septum was detected in two patients that presented a wide lesion comprising almost the whole nasal pyramid; one of them also presented a marginal infiltration of the nasal bones.

The surgical resection of the recurrent lesion was preoperatively planned with a healthy margin of at least 1 cm in all the patients. A wider margin was used whenever needed to include in the surgical resection also the scarring tissue from previous operations, the radiation-damaged tissue, and the potential way of spread of the recurrent lesion. Intraoperative frozen section histopathologic evaluation of the infiltration of the margins was performed in all the cases. Since intraoperative histologic evaluation of the bone margins cannot be assessed, a wider bone resection was performed in the two patients that presented a radiologically confirmed infiltration of the nasal bones and/or skeletal portion of the nasal septum. 

**Figure 1:**
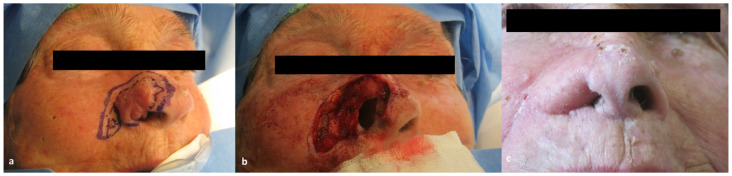
Case of a 79-year-old patient with a post-surgical recurrence of SCCNV at the level of the right ala nasi who underwent surgical resection and immediate reconstruction with a superior-pedicle nasolabial flap. (**a**) Pre-operative picture showing the recurrent lesion on the nasal right ala; (**b**) full-thickness defect after surgical removal; and (**c**) post-operative picture at 6 months of follow-up.

Eleven patients presented a recurrence of SCCNV on the ala nasi. Surgical resection resulted in a full thickness defect of the entire subunit. Immediate reconstruction with a nasolabial flap with a superior pedicle was performed (Figure 1).

**Figure 2:**
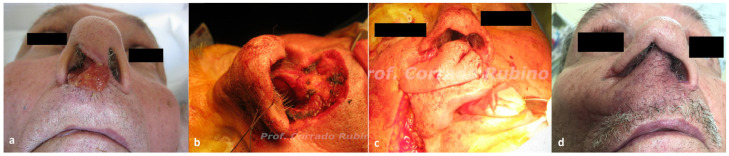
Case of a 70-year-old patient with a post-surgical recurrence of SCCNV at the level of the nasal sill who underwent surgical resection and immediate reconstruction with a superior-pedicle nasolabial flap. (**a**) Pre-operative picture showing the recurrent lesion on the nasal right sill; (**b**) defect after surgical removal comprising full thickness removal of the columella; (**c**) intraoperative picture of the superiorly pedicled nasolabial flap; and (**d**) post-operative picture at 6 months of follow-up.

**Figure 3:**
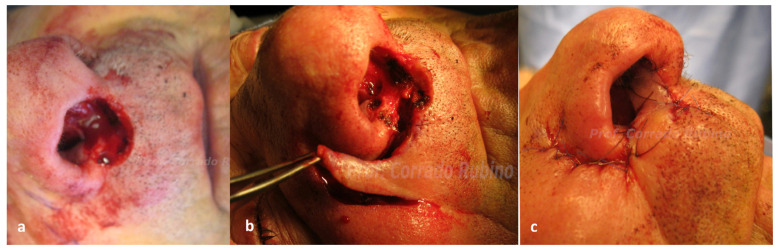
Case of a 68-year-old patient with a post-surgical recurrence of SCCNV at the level of the nasal sill who underwent surgical resection and immediate reconstruction with a peri-alar flap. (**a**) Defect after surgical removal; (**b**) intra operative picture of the perialar flap; and (**c**) immediate post-operative picture.

Three patients had a recurrence at the nasal sill/columella level, with no skeletal infiltration. One patient required a wide excision comprising full thickness removal of the columella. Immediate surgical reconstruction with a nasolabial flap with a superior pedicle (Figure 2) was performed; the flap was folded to recreate the two linings of the columella. One patient underwent recurrence excision comprising nasal sill and homolateral columella lining. In this case, reconstruction with an inferiorly based nasolabial flap was performed. The last case required a smaller resection of the unilateral nasal sill, thus surgical reconstruction with a peri-alar flap was performed (Figure 3). 

**Figure 4:**
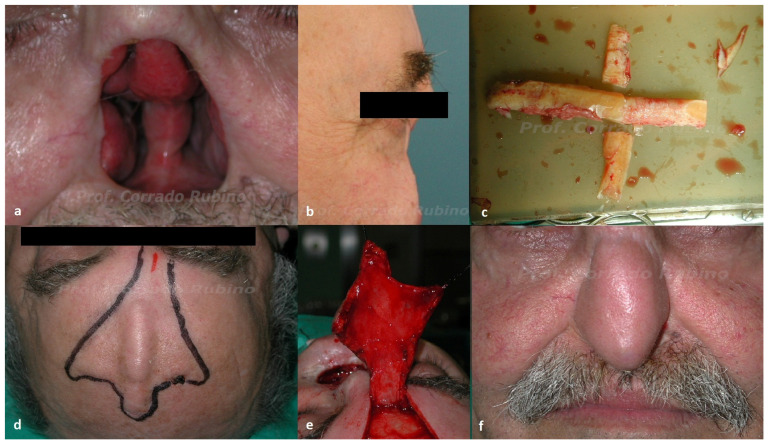
Case of a 61-year-old patient with a post-surgical recurrence of SCCNV at the level of the nasal pyramid with infiltration of nasal cartilages (including the nasal septum) who underwent total rhinectomy and delayed reconstruction with a pre-laminated paramedian forehead flap. (**a**,**b**) Pre-operative picture showing the defect; (**c**) intra operative picture of the cartilaginous graft; (**d**) pre-operative picture of the second reconstructive step showing the prelaminated flap; (**e**) elevation of the pre-laminated flap; and (**f**) post-operative pictures at 24 months of follow-up.

Four patients presented the recurrence at the level of nasal tip/dorsum: they all underwent subtotal nasal resection as infiltration of multiple nasal cartilaginous structures was detected in all cases. The two patients that presented a wide lesion comprising almost the whole nasal pyramid with skeletal infiltration, instead, underwent total rhinectomy. All of these patients underwent surgical reconstruction with paramedian forehead flap. In three cases, structural support was needed and, therefore, we used a prelaminated flap (Figure 4) [22]. In these cases, the first reconstructive step consists of pre-laminating the flap with costal cartilages (Figure 4c), which are inserted between the skin and the galea plane of the forehead in a position corresponding to the future dorsum and columella (Figure 4d). Three to four weeks later, the flap is elevated (Figure 4e) and transferred to the final position, while modeling of the cartilaginous grafts allows to recreate the columella and a physiological tip-projection. Subsequent resection of the pedicle with minor refinements is performed in the final step after an additional four weeks. Resurfacing of the internal lining is obtained through a spontaneous reepithelization of the galea of the flap or adding a skin graft during the second step of the procedure. Delayed surgical reconstruction was opted for five patients, whereas immediate reconstruction was performed in one case due to the patient’s request. The median attendance period for the delayed reconstruction was 18 months (the range was 12 to 20 months).

No post-operative complications were detected in our cohort. Minor surgical revisions were performed in nine patients (45%), two of which were performed for nostril stenosis treatment. Partial impairment to nasal breathing was reported by eight (40%) patients one year after the completion of reconstructive procedures.

**Figure 5:**
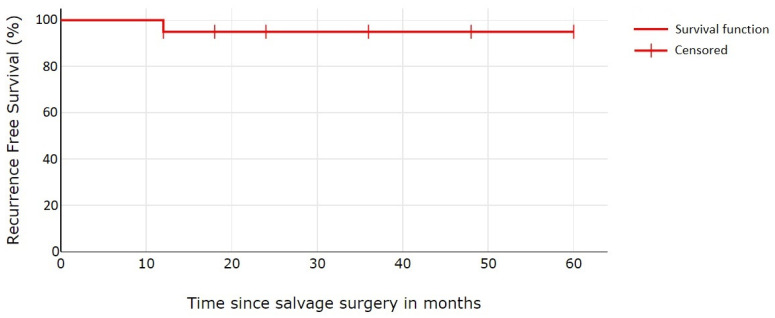
Kaplan–Meier re-recurrence free survival (RFS) estimate for 20 patients with recurrence of SCC of the nasale vestibule after salvage surgical treatment.

The median follow-up period was 36 months (the range was 12 to 60 months). The 5-year RFS estimated with the Kaplan–Meier method for the entire population was 95% (Figure 5). One case of re-recurrence was detected in our cohort. The patient was treated with total nasal resection and immediate reconstruction with the paramedian forehead flap for recurrence of the nasal dorsum with skeletal infiltration. At 12 months post-op, a re-recurrence at the level of the vomer margin was detected during clinical evaluation. He underwent surgical resection of the re-recurrence, but subsequent nose flattening occurred due to partial removal of the skeletal support. The unsatisfactory aesthetic result led the patient to ask for prosthetic reconstruction. 

## 4. Discussion

Local recurrences of nasal vestibule malignancies pose unique challenges in terms of surgical demolition and reconstruction due to the complex anatomy of the region, the pattern of recurrence, and the presence of radiation damage or scars derived from previous treatment of the primary lesion. Based on our experience, pre-operative evaluation should involve an accurate assessment of the recurrence’s size and position. Clinical evaluation should be complemented by a radiological evaluation using CT scan and/or MRI to better assess any potential soft tissue and/or bone infiltration. Residual radiation damage after EBRT or BT and any scars from previous surgeries should be carefully assessed to better determine the amount of tissue that has to be removed and to assess the optimal reconstructive approach. Additionally, it is crucial to consider the peculiar pattern of spread of SCCNV during the surgical planning: this enables the inclusion of the potential way of tumor spread in the surgical resection, increasing the chance of healthy margins and lowering the rate of re-recurrences. Surgical resection with a clinical healthy margin of 1 cm is used in our institution, according to the European Guidelines [23,24] on high-risk cSCC, since disease-specific guidelines are lacking.

In our study group, the male-to-female ratio (4:1) is consistent with the higher prevalence of SCCNV in the male sex reported by other studies [9,21]. Most recurrences occurred after primary surgical treatment (80% of the cases), whereas post radiotherapy recurrences were fewer (20%). This may be attributed to the fact that in the first decade of our experience, our treatment protocol for SCCNV primaries comprised a surgical approach, whereas the use of radiotherapy has been introduced in the last decade. Moreover, in the past, SCCNV were more frequently mistaken as skin primaries, thus requiring multiple surgical resections to obtain complete neoplasm removal. The median period for recurrence appearance detected in our cohort was 17 months, which is in line with the literature data [9,21]. 

The majority of the patients (55%) included in our study showed a recurrence on the ala nasi (Figure 1). In these cases, surgical resection resulted in a full thickness defect of the entire subunit to ensure a clinical margin of 1 cm and to include the potential way of spread, which is usually within the subunit. Since all these patients had undergone prior surgical treatment for the primary lesion, the nearby cheek skin was available as a donor site: a superiorly based nasolabial flap [25,26,27] was therefore used for reconstruction. The nasolabial flap is our preferred choice for full thickness ala nasi reconstruction due to its ease of performance, low donor site morbidity, and favorable aesthetic results. Likewise, the nasolabial flap represents our preferred choice for nasal sill defects reconstruct. Three patients in our cohort, previously surgically treated for the primary lesion, presented a recurrence at the nasal sill. Two patients required resection of the unilateral subunit, and an inferiorly based nasolabial flap (Figure 3) was chosen to cover the defect as it allows to cover the defect without any rotation of the pedicle. One patient, instead, required a wide excision comprising full thickness removal of the columella. In this case, we used a nasolabial flap with a superior pedicle (Figure 2), as it allows to harvest a bigger amount of tissue at the distal portion of the flap that can be folded to recreate the two linings of the columella. Four patients showed a recurrence at the level of nasal tip/dorsum, necessitating subtotal nasal resection due to infiltration of multiple nasal cartilaginous structures. Surgical reconstruction with a paramedian forehead flap was opted for these patients as they were all previously treated with radiotherapy for the primary lesion. The use of local flaps in such cases would have been associated to a higher rate of wound healing problem, whereas the use of a regional flap lowers that risk. Our preferred regional flap for nasal reconstruction is the forehead flap, as it allows to reconstruct the whole nasal pyramid with a perfect skin match, has a low donor site morbidity, and can also be prelaminated with costal cartilages whenever structural support is needed. The last two patients of our study presented a wide lesion comprising almost the whole nasal pyramid with skeletal infiltration. In these cases, total rhinectomy with complete removal of the septum was needed and, therefore, surgical reconstruction with a prelaminated paramedian forehead flap was performed (Figure 4).

Local recurrences of SCCNV are exceptionally rare; therefore, evidence regarding the best treatment option for local recurrences is lacking. Several authors have used RT and surgery, either independently or in combination, as salvage treatment [5,6,20,21], with favorable rates of locoregional disease control. However, as worse aesthetic outcomes have been reported with the use of BT in case of recurrences [16], surgery remains the current standard in such cases. Based on our experience, the surgical salvage procedure for SCCNV recurrence differs from the treatment of primary cases. The presence of scars from previous surgeries or radiotherapy skin damage entails the necessity of a different approach for both surgical resection and reconstruction. Alongside tumor removal, resection of the surrounding scars and heavily radiotherapy-damaged skin is crucial to ensure viable margins for better wound healing. Radiotherapy profoundly impacts the treated skin, inducing several acute and long-term side effects such as microvascular damage and skin atrophy, leading to a higher rate of wound healing problems [28]. Consequently, in case of previous radiotherapy, surgical resection with a wider-than-1 cm margin may be necessary to include all the heavily damaged tissue. In the case of post-surgery recurrences, instead, resection with a 1 cm margin may be sufficient to remove the surrounding scar tissue. Reconstruction with local flaps is feasible in these patients, given the viability of the surrounding tissue. As shown in our study cohort, the nasolabial flap emerges as our preferred local flap due to its versatility in the reconstruction of a wide range of dimensions and location of nasal defects. Similarly, in the cohort reported by Horsman et al. [5], the nasolabial flap was the most used reconstructive option for local recurrences. Alternative options for reconstructing the nasal sill include the Abbè flap [29,30], alone or combined with the paramedian forehead flap, and the crab-pincers style facial artery (CPFA) flap [31], according to the excision’s extension. Previous radiotherapy has a huge impact on the reconstructive planning too, since the use of local flap derived from the surrounding radiated tissue, including mucosal flaps, is not recommended due to the higher rate of wound dehiscence, infection, and flap necrosis [32,33]. This is especially true with primary EBRT, since BT seems to have lower impact on surrounding tissue [12,15]. In such cases, regional flaps derived from farther areas of the face or free flaps are preferred. The paramedian forehead flap is our preferred option in case of previously radiation-treated patients and whenever the dimension of the defect cannot be reconstructed with local flaps. In our cases, the internal lining resurfacing was obtained through spontaneous reepithelization of the galea of the flap or adding a skin graft during the second step of the prelamination procedure. Previous radiotherapy or the absence of sufficient tissue in total rhinectomy cases precluded the use of mucosal flaps to recreate the inner nasal lining. On the contrary, resurfacing of the internal lining in primary cases is possible with mucosal flaps, as described by different authors [3,7]. Conjunct reconstruction with free fascial or fascio-cutaneous flaps for interior lining in combination with a paramedian forehead flap to reconstruct the nasal skin is another viable option [34,35]. In particular, the use of free radial forearm fascial of the fascio-cutaneous flap [36,37] and nonlaminated free temporal fascial flap [38] has been described in the literature. In addition to the forehead flap, the reverse submental flap [39] has been described in the literature as a viable mean for nasal sill/columella reconstruction; the flap can also be prelaminated with costal cartilages for columellar support reconstruction. Free flaps, instead, represent the last resource in the case that the paramedian forehead flap has already been used or in the case that the patient does not want any adjunctive scar on the face. The forearm free flap [40], anterolateral thigh free flap [41], and ulnar free flap [42] have been described in the literature for subtotal/total nasal reconstruction. These flaps can be prelaminated with cartilaginous grafts, as described for the paramedian forehead flap, for framework reconstruction. Skeletal reconstruction with the medial femoral condyle free flap [43] or osteo-cutaneous radial forearm free flap [44] in combination with a paramedian forehead flap has been also reported in literature. An alternative to regional and free flaps use in subtotal rhinectomy cases is the use of a custom-made nasal prosthesis [45,46]. Implantation offers the clear advantage of reduced operative time; this is especially important in elderly or fragile patients, for whom multiple-steps reconstructive surgery may be complicated or even contraindicated. The use of removable prosthetic also enables high-quality postoperative monitoring for re-recurrences. Moreover, the esthetic result is favorable, with a positive impact on patients’ psychosocial well-being and quality of life [47,48]. Nevertheless, prior radiotherapy might negatively affect implantation, even though literature data regarding implant survival at the nasal site are controversial [49]. The accurate selection of the implant location, time of implantation, and loading protocol should be carefully evaluated according to the patients and radiotherapy characteristics [50,51]. 

The intraoperative histopathological assessment of healthy margins is routinely performed in our unit during salvage procedures. Since intraoperative assessment of skeletal margins is not possible, we advocate for considering a delayed reconstruction in case of recurrences involving deep skeletal structures. This recommendation extends to cases with infiltration of the deep portion of the cartilaginous nasal septum, where early detection of recurrences at the level of the deep margins is more difficult in case of immediate reconstruction. The flap would, in fact, cover the recurrent lesion, making the clinical detection of a potential recurrence more difficult. Delayed reconstruction after at least 12 months of follow-up after recurrence resection is thus routinely proposed in our unit to all patients with extended lesion with cartilaginous and/or skeletal infiltration that require complex reconstruction. According to Teichgraeber and Goepfert [52], the site of the lesion is significant in predicting recurrence, whereas histology and tumor size are significant predictors of the need for a total or subtotal rhinectomy. Consequently, they recommend a delayed reconstruction after 2 years in patients with poor general status and in the case of large recurrent lesions that are multicentric or involve the ala or dorsum; prosthetic rehabilitation is also suggested as a good interim measure until the time of reconstruction. The described approach allows us to save the best reconstructive option for a delayed reconstruction, after complete removal of the recurrence has been confirmed by the histopathological analysis and a clinical surveillance for re-recurrences has been performed for a fair number of months. In our case series, we reported one case of re-recurrence (5%). The patient presented skeletal infiltration, hence surgical resection and delayed reconstruction were proposed to him as salvage treatment. Due to psychological issues, the patient requested immediate reconstruction, which was performed with a forehead pre-laminated flap. At a 12 months post-operative follow-up, a re-recurrence was detected during a rhinoscopy, leading to surgical resection without any adjunctive reconstruction. As a result, nose flattening occurred with an unsatisfactory aesthetic result, and the patient underwent prosthetic reconstruction. Recurrences are documented to occur within 8 to 42 months post-treatment [4,5,9,20] in literature. Patients with a history of SCCNV should therefore be followed closely, especially during the first few years after diagnosis. There are no standardized follow-up guidelines for patients with SCCNV, and the length of follow-up is usually dictated by institutional protocols. 

**Figure 6:**
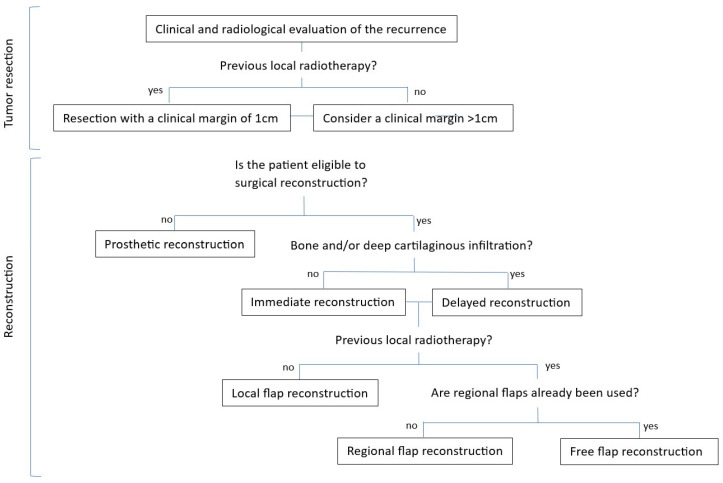
Schematic representation of our salvage strategies for local recurrences of squamous cell carcinoma of the nasal vestibule.

The presented strategy combines oncologic resection and nasal reconstruction (Figure 6). Surgical resection comprising the potential way of tumor spread is a crucial element in our strategy, aiming for higher chances of healthy margins and lower rates of re-recurrence. The resection of the surrounding scars and heavily radiotherapy-damaged skin is also fundamental to ensure viable margins with better wound healing and lower rate of post-operative complications, especially in the case of immediate reconstruction. Reconstruction after SCCNV recurrence resection is a great challenge for the plastic surgeon, but it is feasible with available local, regional flaps, or free tissue transfer, as shown in this article. Prosthetic nasal reconstruction, instead, is particularly important in elderly or fragile patients, for whom multiple-steps reconstructive surgery may be complicated or even contraindicated. Finally, delayed reconstruction must be considered whenever the patient presents an extended lesion with cartilaginous and/or skeletal infiltration, as intraoperative assessment of the margins is not possible, and early detection of a recurrences at the level of the deep margins would be more difficult in the case of immediate reconstruction as the flap would cover the recurrent lesion. 

The limitations of this paper include the retrospective design of the study and the small study group. Data regarding patient-reported satisfaction and quality of life are missing since some cases date back more than 20 years, whereas a structured evaluation of these outcomes has been introduced in our institution only in the last decade.

## 5. Conclusions

On the bases of our experience, salvage surgical procedure for SCCNV recurrences must be individualized and carefully planned, taking into account the peculiar pattern of spread of such recurrences, the presence of damaged tissue from previous treatment, the location of the recurrence, and the infiltration of surrounding tissue.

## Figures and Tables

**Table 1:** Demographic, local recurrence, and surgical characteristics of the study group (20 patients).

Variable		No.	Percentage
Age	Median	71	
	Range	56–78	-
Gender	Male	16	80%
	Female	4	20%
Primary lesion staging (Wang Staging System)	T1	11	55%
	T2	8	40%
	T3	1	5%
Primary lesion G (grading)	G1	1	5%
	G2	16	80%
	G3	3	15%
Primary lesion treatment	Surgical	16	80%
	EBRT	3	15%
	BT	1	5%
Period for recurrence appearance (months)	Median	17	
	Range	8–30	-
Subsite of recurrent lesion	Nasal alae	11	55%
	Nasal tip/dorsum	6	30%
	Columella/nasal sill	3	15%
Reconstructive procedure	Nasolabial flap with a superiorly based pedicle	12	60%
	Nasolabial flap with a inferiorly based pedicle	1	5%
	Peri-alar flap	1	5%
	Paramedian forehead flap	6	30%
Surgical revision		9	45%
Follow-up (months)	Median	36	
	Range	12–60	
Re-recurrence		1	5%

EBRT External Beam Radiotherapy; BT Brachytherapy.

## Data Availability

The data used and/or analyzed during the current study are available from the corresponding author upon reasonable request.
